# Protocol to determine antibody affinity and concentration in complex solutions using microfluidic antibody affinity profiling

**DOI:** 10.1016/j.xpro.2023.102095

**Published:** 2023-02-13

**Authors:** Marc Emmenegger, Roland Worth, Sebastian Fiedler, Sean R.A. Devenish, Tuomas P.J. Knowles, Adriano Aguzzi

**Affiliations:** 1 Institute of Neuropathology, University of Zurich, 8091 Zurich, Switzerland; 2 Fluidic Analytics, Unit A, The Paddocks Business Centre, Cherry Hinton Road, Cambridge CB1 8DH, UK; 3 Centre for Misfolding Diseases, Yusuf Hamied Department of Chemistry, University of Cambridge, Lensfield Road, Cambridge CB2 1EW, UK; 4 Cavendish Laboratory, Department of Physics, University of Cambridge, JJ Thomson Avenue, Cambridge CB3 0HE, UK

**Keywords:** Surface Plasmon Resonance (SPR), Health Sciences, Immunology, Antibody, Protein Biochemistry, Proteomics

## Abstract

Conventional methods of measuring affinity are limited by artificial immobilization, large sample volumes, and homogeneous solutions. This protocol describes microfluidic antibody affinity profiling on complex human samples in solution to obtain a fingerprint reflecting both affinity and active concentration of the target protein. To illustrate the protocol, we analyze the antibody response in SARS-CoV-2 omicron-naïve samples against different SARS-CoV-2 variants of concern. However, the protocol and the technology are amenable to a broad spectrum of biomedical questions.

For complete details on the use and execution of this protocol, please refer to Emmenegger et al. (2022),^1^ Schneider et al. (2022),^2^ and Fiedler et al. (2022).^3^

## Highlights

• Step-by-step protocol detailing microfluidic antibody affinity profiling
• Measurement of antibody affinity in solution on an automated system
• Determination of affinity in non-omicron plasma to SARS-CoV-2 variants of concern
• Broad applicability in science and medicine, including immunology and drug development

## Abstract (editor highlights)

Publisher’s note: Undertaking any experimental protocol requires adherence to local institutional guidelines for laboratory safety and ethics.

## Graphical abstract

**Figure:**
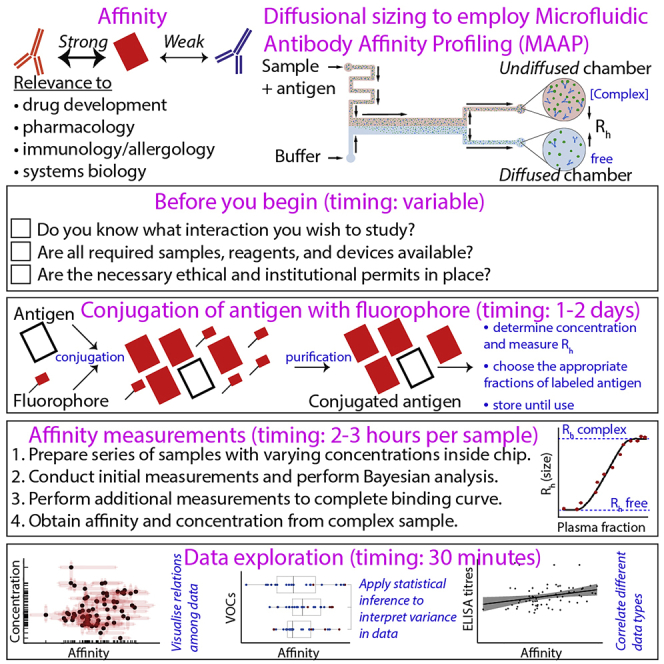

## Before you begin

**State of art and usefulness of the protocol for the community**: On the molecular level, interactions are characterized by two properties: affinity (i.e., the strength of binding) and concentration.^1^ The information of the respective affinity of a molecule for various other molecules is a direct measure of specificity. For instance, a molecule with high affinity for multiple other molecules might be considered less specific than a molecule that interacts only with one other molecule at high affinity. The gold standard for experimentally assessing affinity is surface plasmon resonance (SPR); however, SPR has important limitations: (1) although SPR has been successfully employed to assess the interaction of a monoclonal antibody to its target antigen, the method is unsuitable to determine affinity directly in biological samples such as in blood. (2) SPR and similar technologies – for instance biolayer interferometry (BLI) or enzyme-linked immunosorbent assay (ELISA) – rely on the immobilization of either the antibody or the antigen, inducing well-known surface effects.^4^^,^^5^ The protocol presented here details a reliable, automated, simple, and user-friendly method to determine the affinity of a molecular interaction *in solution* using complex samples like plasma. This is advantageous as it allows one to track for instance the antibody response in patients over time following the administration of therapeutic antibodies (immunotherapy), to determine the presence of autoantibodies in autoimmune disease, to pharmacologically assess the potency of a monoclonal antibody following its generation, or to infer fundamental biophysical properties (i.e., affinity and concentration) of almost any protein-protein interaction in a standardized manner.

**Relevance and amenability of the specific protocol presented here**: The protocol below describes the specific steps required to perform affinity measurements in plasma or serum samples of patients whose humoral immune response is likely polyclonal, however, certain clones may dominate the response; with simulations indicating that the measured antibody response is usually attributed to the concentration of the strongest, i.e., most affine, binder.^2^ Although this protocol specifically focuses on measurements in plasma or serum, all the steps are amenable to studying almost any kind of protein-protein interaction, including monoclonal antibodies to specific antigens, for instance aggregated α-synuclein,^6^ commercial anti-amyloid β antibodies,^7^ or monoclonal antibodies directed against an antigen of interest.^2^^,^^3^ Before the protocol starts, it is worth clarifying the key requirements.

### What interaction do you want to study?

**Timing: variable**

1. Be clear about the interaction you wish to study, e.g., the affinity of a monoclonal antibody to a particular antigen. In this protocol, as a practical example, we measured the affinity and active concentration of antibodies against the wild type, delta, and omicron VOC of the SARS-CoV-2 receptor binding domain (RBD) protein in the blood of individuals post infection and/or post vaccination.

***Note:*** Throughout the protocol we refer to experimental protocols used to generate the data, the code used for the analysis of some of the data, and to a previously published dataset containing MAAP measurements performed with 39 patients with a history of infection with SARS-CoV-2 and/or vaccination against SARS-CoV-2 and two control individuals without SARS-CoV-2 infection/vaccination^1^ as a practical example.

### Clarify the availability of the reagents you require

**Timing: variable**

2. Accordingly, make sure that the reagents are available, e.g.
  a. The monoclonal antibody of choice, or in our case the plasma or serum samples. To obtain a complete serological fingerprint ∼50 μL of undiluted serum is required to perform a MAAP assay that will determine both the affinity and concentration against a target protein.
  b. The antigens of choice, which in this protocol are the RBD for three SARS-CoV-2 variants of concerns (VOC).

### Clarify the availability of the devices you require

**Timing: variable**

3. Affinity measurements can be performed on microfluidic chips by using confocal fluorescence microscopy for the readout (see e.g.^6^). In this protocol, we will be referring to an *automated version of this assa*y, available as the Fluidity One-M (Fluidic Analytics, Cambridge, England). To perform affinity measurements using this protocol, access to the Fluidity One-M device needs to be granted either through your own lab or via a core facility. If a machine is not available in your neighborhood, Fluidic Analytics offers an analytical service via its Protein Interactions Lab (PIL) where one can send samples for analysis following the steps detailed in this protocol.

***Note:*** The measurements conducted using the Fluidity One-M instrument rely on a biophysical principle called microfluidic diffusional sizing (MDS) and the application of this technology has been described in depth elsewhere.^8^^,^^9^ In brief, during MDS, two fluid streams run alongside one another with no convective mixing in a microfluidic channel and therefore the diffusion of a particle lateral to the direction of flow is governed only by the size, or hydrodynamic radius (*R*_h_), of the particle (see top right panel in the Graphical abstract). In this context, small particles (e.g., an unbound labelled antigen) will diffuse more rapidly than a labelled antigen in complex with its interacting partner (e.g., an antibody). By measuring the *R*_h_ of a molecule with MDS, when in the presence of its interacting partner, an increase in size will correlate with a binding event.

### Clarify the availability of necessary ethical and institutional permits

**Timing: variable**

4. When using biospecimens (e.g., blood, urine, tracheobronchial secretion, saliva, cerebrospinal fluid) of human donors, ensure to obtain the necessary ethical and institutional permits well in advance.

### Institutional permissions

For this protocol whose scientific results have been previously published,^1^ we included residual pre-omicron heparin plasma samples from 41 patients (median age 65 (interquartile range (IQR): 54–77) years; distribution of female-male sex 0.41:0.59) admitted to the University Hospital Zurich, Zurich, Switzerland, whose blood was sent to the Institute of Clinical Chemistry of the same hospital for routine diagnostic procedures. All experiments and analyses involving samples from human donors were conducted with the approval of the ethics committee of the canton Zurich (KEK Zürich), Switzerland (KEK-ZH-Nr. 2015–0561, BASEC-Nr. 2018–01042, and BASEC-Nr. 2020–01731), in accordance with the provisions of the Declaration of Helsinki and the Good Clinical Practice guidelines of the International Conference on Harmonisation. All subjects enrolled in the study signed the hospital-wide general consent of the University Hospital Zurich, Switzerland.

## Key resources table

**Table:** 

REAGENT or RESOURCE	SOURCE	IDENTIFIER
**Biological samples**		

University Hospital Zurich plasma samples	iScience article^1^	https://doi.org/10.1016/j.isci.2022.104766

**Chemicals, peptides, and recombinant proteins**		

Wild Type SARS-CoV-2 RBD, HEK293	Sino Biological	40592-V08H
Delta SARS-CoV-2 RBD, HEK293	Sino Biological	40592-V08H90
Omicron SARS-CoV-2 RBD, HEK293	Sino Biological	40592-V08H121
Alexa Fluor™ 647 NHS ester	Thermo Scientific	A20006
Software		
Fluidity Cloud	Fluidic Analytics	N/A
*R 4.2.0* statistical software	R Core Team	N/A
*R Studio 2022.07.1 Build 554*	R Studio, PBC	N/A
Code employed in this study	Zenodo^10^	https://doi.org/10.5281/ZENODO.7455494

**Other**		

ÄKTA™ Pure Protein Purification System	Cytiva	N/A
1 mL 7k MWCO Zeba™ chromatography desalting cartridge	Thermo Scientific	89934
10 kDa Amicon® Ultra Centrifugal Filter	Merck	UFC5010
Fluidity One-M instrument	Fluidic Analytics	N/A
Fluidity One-M chip plate	Fluidic Analytics	N/A
Nanodrop One	Thermo Scientific	N/A

## Materials and equipment

**Table:** 1 M labeling buffer solution

Reagent	Final concentration	Amount
NaHCO_3_	1 M	8.4 g
ddH_2_O	N/A	95 mL
HCl	N/A	until pH 8.3
ddH_2_O	N/A	ad 100 mL
**Total**	**N/A**	**100 mL**

**Table:** Viscomatch buffer

Reagent	Final concentration	Amount
Glycerol	10% w/v	5 g
PBS, pH 7.4	N/A	15 mL
HSA	5% w/v	2.5 g
PBS, pH 7.4	N/A	ad 50 mL
**Total**	**N/A**	**50 mL**

## Step-by-step method details

### Conjugation of protein with fluorescent label

**Timing: 1–2 days**

**Figure 1:**
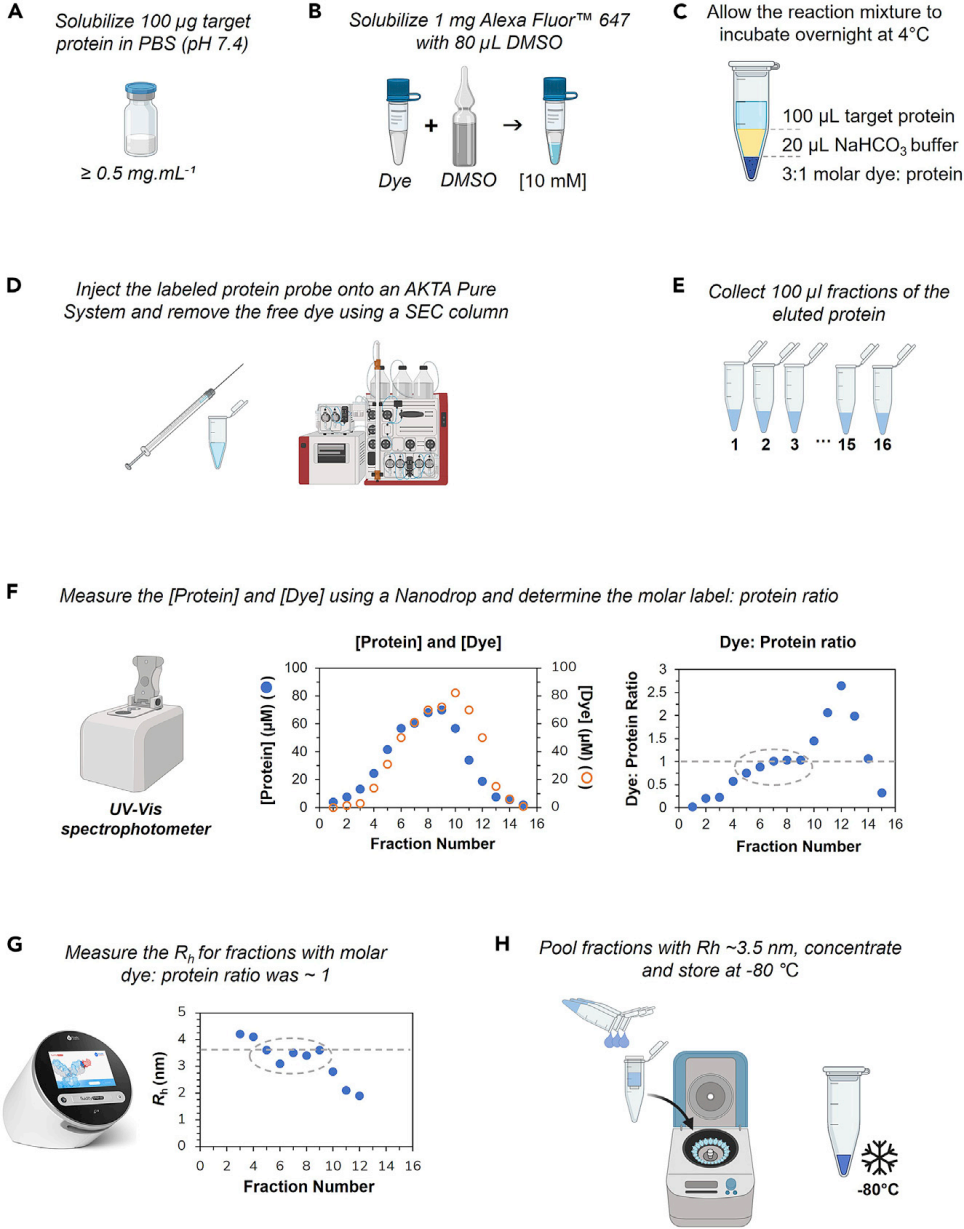
Workflow to effectively conjugate an antigen with Alexa Fluor™ 647 for use in a MAAP assay that will determine the affinity and concentration of the labeled antigen to its target antibody (A-C) The target protein is solubilized in PBS and conjugated with Alexa Fluor 647 dye. (D-F) Purification is conducted on an AKTA Pure System, fractions of the eluted Alexa 647-conjugated target protein are collected, and the molar label:protein ratio is calculated on a Nanodrop. (G-H) For those fractions for which the dye:protein ratio is close to 1, the *R_h_* is measured (G). The fractions where *R_h_* is close to 3.5 nm are pooled, concentrated, and stored.

The first critical step to determining the affinity of an antibody to the target antigen of interest (e.g., the SARS-CoV-2 RBD used in this study) is the proper conjugation of the antigen with the correct fluorophore. Here, we detail all the steps required to obtain a high-quality labeled antigen (schematically summarized in Figure 1).

1. Dissolve 1 mg of Alexa Fluor™ 647 NHS ester in 80 μL of dimethyl sulfoxide (DMSO) to prepare a 10 mM solution.

**CRITICAL:** Due to the hygroscopic properties of DMSO, always ensure to use a fresh, unopened vial of DMSO and do not re-use once open. Instead, prepare 100 μL aliquots of DMSO and store at −20°C, but do not re-freeze once it has been thawed.

2. Dissolve 8.4 g of sodium bicarbonate (NaHCO_3_) in 95 mL of ultrapure water and adjust the pH to 8.3 using 1 M HCl. Thereafter, add ultrapure water to a final volume of 100 mL to prepare a 1 M labeling buffer solution.

***Note:*** The storage temperature of the labeling buffer solution is at −20° Celsius and its storage up to 180 days or longer.

3. Using 100 μg of the protein probe (e.g., SARS-CoV-2 RBD), solubilize the protein to a minimum concentration of 0.5 mg · mL^−1^ in PBS buffer (pH 7.4).

**CRITICAL:** If the protein probe is dissolved in a buffer which contains primary amines (e.g., Tris) make sure to exchange into PBS buffer prior to labeling. This can be achieved by using a Zeba™ chromatography desalting cartridge.

4. Add the 1 M labeling buffer to the protein probe solution to a final concentration of 200 mM by adding 20 μL of the labeling buffer to 100 μL of the protein solution.
5. Then add the Alexa Fluor™ label stock solution to the protein probe at a molar label:protein ratio of 3:1 and allow the labeling reaction to incubate overnight at 4°C.

***Note:*** Considering that the label is dissolved in DMSO, ensure that the final concentration of the label stock solution in the label reaction does not exceed 1.8% (v/v) otherwise the DMSO could interfere with the structural integrity of the probe. Moreover, to avoid photobleaching of the dye ensure to keep the reaction mixture in a dark environment.

6. Using a Superdex 75 Increase 10/300 column (Cytiva) that is coupled to an ÄKTA™ Pure Protein Purification System (Cytivia) pass the reaction mixture through the column to separate the free Alexa Fluor™ 647 dye from the labeled protein solution by size-exclusion chromatography (SEC) and collect 100 μL fractions of the eluted protein.

***Note:*** Depending on the purity or homogeneity of the protein sample the free dye can alternatively be removed from the protein sample by using a Zeba™ desalting column (Thermo Scientific). However, the use of a SEC column is advantageous to simultaneously remove the free dye and purify the protein as a polishing step.

7. Using a Nanodrop One (Thermo Scientific) determine the concentration of both the protein probe and conjugated label in each fraction using a wavelength of 280 nm and the Alexa Fluor™ 647 setting on the instrument, respectively.

***Note:*** When measuring the protein concentration ensure that the sloping dye correction and analysis correction function on the Nanodrop are disabled.

8. Based on the measured protein yield (in mg · mL^−1^), convert the protein concentration to molarity and determine the labeling ratio by looking at the protein: dye concentration ratio.

***Note:*** For globular proteins such as RBD, the molecular weight can be converted to the hydrodynamic radius assuming the protein adopts a compact, globular fold using the online calculator tool provided here, or a PDB structure can be used to predict a hydrodynamic radius using the online tool provided here.

**CRITICAL:** Some proteins carry posttranslational modifications and do therefore have a larger hydrodynamic radius when measured by MDS. While the expected *R*_h_ based on the nominal molecular weight of RBD would be 2.5 nm, it is experimentally measured to have 3.5 nm due to glycosylation.

9. The Fluidity One-M instrument measures the hydrodynamic radius (*R*_h_) of the fractions with labeling ratios of 0.5–2. The measured *R*_h_ – here – should be around 3.5 nm.
10. Pool those fractions with a measured *R*_h_ ∼ 3.5 nm, concentrate the sample using a 10 kDa Amicon® Ultra Centrifugal Filter unit and measure the final protein and dye concentration using a Nanodrop One (Thermo Scientific). The final concentrated and labeled sample should be stored at –80°C in PBS (pH 7.4) containing 10% (w/v) glycerol as a cryoprotectant.

### Affinity and concentration determination using Fluidity One-M

**Timing: 2–3 h per sample and antigen**

**Figure 2:**
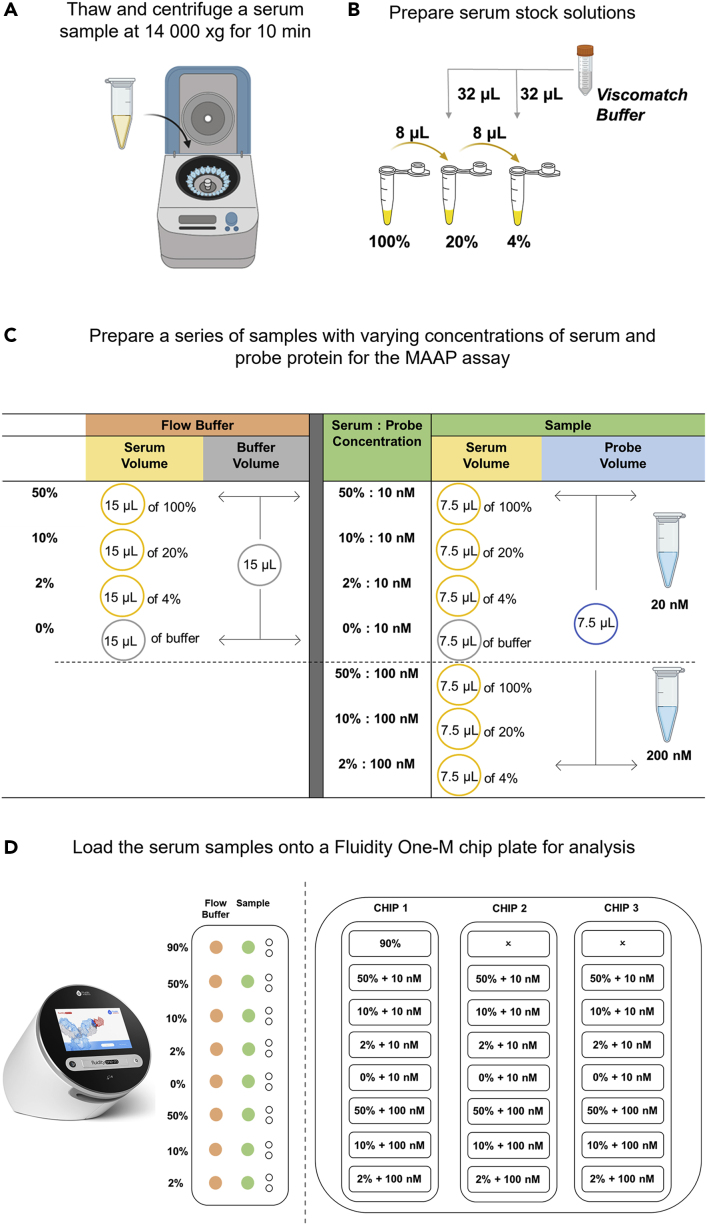
Workflow for preparing serum samples for a MAAP assay that will be analyzed by MDS on the Fluidity One-M (Fluidic Analytics) (A) A serum or plasma sample is thawed and centrifuged at 14,000 xg for 10 min. (B) The serum stock samples are prepared using Viscomatch buffer. (C) The flow buffer dilutions and serum samples containing the probe protein are prepared. (D) The Fluidity One-M chip plate is loaded for analysis.

First prepare the buffer solution used in the protocol (Figure 2A).

11. To prepare a viscomatch buffer (PBS, 5% (w/v) human serum albumin (HSA) and 10% (w/v) glycerol, pH 7.4), weigh 5 g of glycerol and dissolve in ∼ 15 mL of PBS (pH 7.4). Transfer the resuspended glycerol to a 50 mL falcon tube containing 2.5 g of HSA and add PBS (pH 7.4) to a final volume of 50 mL. Allow the HSA to dissolve by slowly inverting the mixture overnight at 4°C.

***Note:*** The storage temperature of the viscomatch buffer is at 4° Celsius and its storage up to 30 days or longer.

The next step is to prepare the patient samples (Figure 2B).

***Note:*** In the study presented in this protocol, heparin plasma samples were used. However, the method works equally with serum samples. For the sake of readability of the protocol, we refer to plasma or serum interchangeably.

12. Thaw a sample of human plasma or serum and centrifuge at 14,000 × *g* for 10 min at 4°C and decant the supernatant to a fresh Eppendorf tube for subsequent use.
13. Take 9 μL of undiluted (100%) serum and mix it with 1 μL of the viscomatch buffer to prepare a serum solution at a final concentration of 90%.
14. Take 8 μL of the 100% serum and mix it with 32 μL of the viscomatch buffer to prepare a 20% serum solution.
15. Then take 8 μL of the 20% serum and mix it with 32 μL of the viscomatch buffer to prepare a 4% serum solution.

Prepare the stock solutions of the conjugated RBD VOCs and mix them with the patient serum samples (Figure 2C).

16. Prepare 100 μL of a 20 nM and 200 nM stock solution of the probe protein, here conjugated SARS-CoV-2 wild type, delta, or omicron RBD variants, in the viscomatch buffer.

***Note:*** To keep this part of the protocol as generic as possible, we here refer to the probe protein rather than specifically to conjugated RBD VOCs.

17. To prepare the final samples, mix the stock solutions of the probe protein and the samples at equal amounts with each other. In detail, take 7.5 μL of the 4, 20 and 100% serum and mix each sample with 7.5 μL of the 20 nM protein probe stock solution to prepare samples of 0, 2, 10 and 50% serum in the presence of 10 nM of the probe protein.
18. Repeat the same process to prepare samples with 100 nM of labeled probe by using the 200 nM protein probe stock solution.

***Note:*** Here, we use a low concentration of probe protein (i. e., 10 nM) to detect binding of low concentration targets contained in the sample. This approach is further explained in the supplemental information of work recently published.^1^

19. Prepare a similar set of samples with matching buffer (i.e., serum flow buffer) by taking 15 μL of the 4, 20 and 100% serum and mixing it with 15 μL viscomatch buffer.
20. Incubate the samples on ice or a cooling block at 4°C for a period of 30 min.

Having prepared the samples, load them onto a Fluidity One-M microfluidic chip (Figure 2D).

21. Using the Fluidity Cloud platform (Fluidic Analytics), create a custom MAAP assay template for the Fluidity One-M chip plate layout. Fill in the details of each sample such as the serum concentration, protein probe concentration and buffer composition for each circuit on the plate. For this study a wavelength of 647 nm (i.e., red wavelength), a viscosity setting of 3 and a size range of 3 (3–14 nm) will need to be selected.

***Note:*** The viscosity setting of 3 is the default setting for serum and plasma but other probes or biospecimen may have a different viscosity property. The size range described here is characteristic of the SARS-CoV-2 experiment performed to demonstrate the assay. For other types of samples, sizes may vary. Fluidic Analytics provides a tool to predict sizes based on molecular weights as a guidance for selection of appropriate size ranges.

22. Using a Fluidity One-M microfluidic chip plate (Fluidic Analytics) load 4 μL of the 90% serum in both the auxiliary and sample port in circuit A1 to determine the maximum background signal of the sample (Figure 2D).
23. Load 4 μL of the 50% serum flow buffer in the auxiliary ports of circuits B1, 2, and 3 and F1, 2, and 3, 4 μL of the 10% serum flow buffer in the auxiliary ports of circuits C1, 2, and 3 and G1, 2, and 3, 4 μL of the 2% serum flow buffer in the auxiliary ports of circuits D1, 2, and 3 and H1, 2, and 3 and 4 μL of the viscomatch buffer in the auxiliary ports of circuits E1, 2, and 3. Allow 90 s for the flow buffer to prime the circuits (Figure 2D).
24. Load 4 μL of the 50% serum reaction mixture in the sample ports of circuits B1, 2, and 3 and F1, 2, and 3, 4 μL of the 10% serum reaction mixture in the sample port of circuits C1, 2, and 3 and G1, 2, and 3, 4 μL of the 2% serum reaction mixture in the sample ports of circuits D1, 2, and 3 and H1, 2, and 3, and 4 μL of the 0% serum sample in circuits E1, 2, and 3. As highlighted in Figure 2D, circuits B-E will contain 10 nM of the protein probe and circuits F-H will contain 100 nM of the protein probe.

***Note:*** The settings referred to above are suitable for SARS-CoV-2 RBD VOCs. Other proteins of other molecular sizes may require different parameters.

25. Load the template (.csv file) onto the Fluidity One-M (Fluidic Analytics) and select “New Experiment”. Then load the chip plate containing the samples of interest into the instrument and select “Start Run”. A full chip plate will take ∼25 min to complete.
26. Once complete export the data (.csv and .json file) and upload the JSON files onto the Fluidity Cloud (Fluidic Analytics) using the “Upload Measurements” tab.
27. Once uploaded set up a “New Experiment” using the “MAAP assay with Bayesian fit”, “Add” the data files, click “Create Experiment” and “Run analysis”.

**Figure 3:**
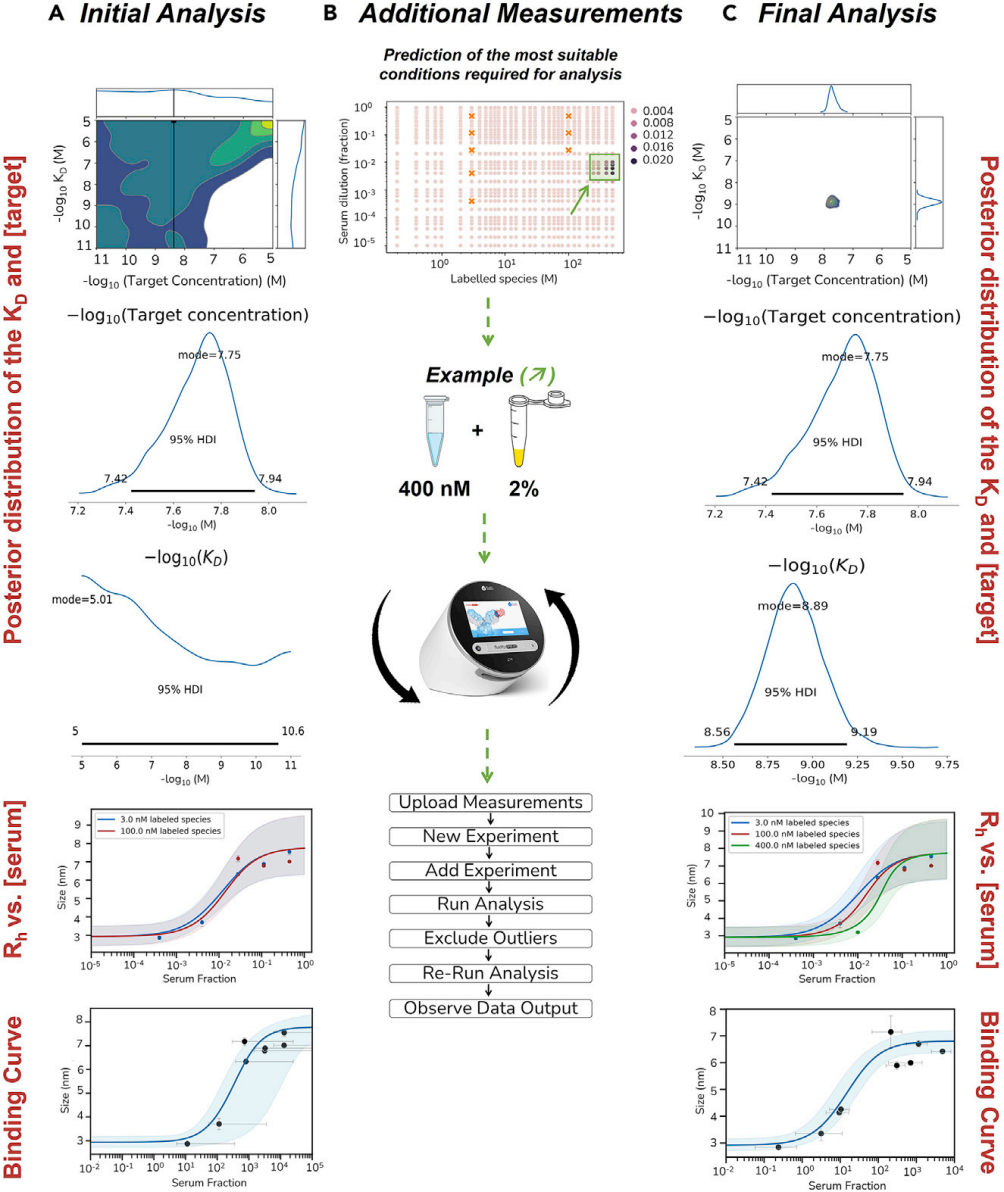
Additional measurements to refine the *K*_D_ and target concentration parameters (A) The data from the initial conditions (i.e., 2, 10 and 50% of serum with 10 and 100 nM of the protein probe) may yield a complete data set. (B) In most situations, additional samples need to be prepared to better refine the data. Using Bayesian statistical analysis, the Fluidity Cloud (Fluidic Analytics) will predict a concentration range of both the serum and protein probe concentration that will refine the data set. (C) The refined data set yielding a defined *K*_*D*_ and target concentration for the interaction after additional measurements.

Based on these measurements, we obtain our first results (Figure 3A).

28. Once the data analysis is complete the results of the Bayesian analysis will be reflected as a summary of the calculated *K*_D_, the target concentration, a binding curve and a summary of the background signal-corrected *R*_h_ and -fluorescence intensity for each measured sample. Furthermore, additional tabs will reflect the quality control of the data analysis and a posterior distribution of the *K*_D_ and target concentration parameters of the model.
29. The data from the initial conditions (i.e., 2, 10 and 50% of serum with 10 and 100 nM of the protein probe) may yield a complete data set, but in many situations additional samples need to be prepared to better refine the data. Using Bayesian statistical analysis, the Fluidity Cloud (Fluidic Analytics) will predict a concentration range of both the serum and protein probe concentration that will refine the data set (Figure 3B). For instance, in Figure 2, based on the predictive concentration range a sample containing 400 nM of the protein probe and 2% of the patient serum was prepared to refine the measured data. The additional samples will be prepared and measured in a similar manner to the steps mentioned previously.

In the current study, we performed a MAAP assay to obtain affinity and concentration measurements in samples of 41 patients. The final *K*_*D*_ and active concentration values were obtained after the iterative measurement approach depicted above (see Figure 3C). In some cases, there were simply no anti-RBD antibodies, or the concentration and/or affinity of the antibody:RBD interaction was too low that no binding event could be measured. In total, the affinity and concentration for antibody:RBD interaction were determined in 67% of samples for wild type RBD, in 64% of samples for delta RBD, and in 51% of samples for omicron RBD. Importantly, the limit of detection (sensitivity) for this method was shown to be at a *K*_*A*_ of around 0.01 nM^-1^ (i.e., *K*_*D*_ of around 100 nM) and at a probe protein concentration of about 10 nM (see supplementary materials in^1^). The data from the MAAP assay for all patients has been summarized in Table 1. Thus, in terms of strict biophysical characterization of our samples, the journey – i.e., the protocol – ends here. The next chapter shows one possible way (among many others) of how affinity and concentration measurements can be used.

**Table 1:** Overview of the data from the MAAP assay measurements

Patient number	*K*_*A*_ wild type	*K*_*A*_ wild type lower 95% CI	*K*_*A*_ wild type lower 95% CI	*K*_*A*_ delta	*K*_*A*_ delta lower 95% CI	*K*_*A*_ delta lower 95% CI	*K*_*A*_ omicron	*K*_*A*_ omicron lower 95% CI	*K*_*A*_ omicron lower 95% CI	Conc. wild type	Conc. wild type lower 95% CI	Conc. wild type lower 95% CI	Conc. delta	Conc. delta lower 95% CI	Conc. delta lower 95% CI	Conc. omicron	Conc. omicron lower 95% CI	Conc. omicron lower 95% CI
026																		
037																		
005	7.23	7.63	6.89	7.32	7.81	6.92				-5.23	-5.55	-4.99	-5.38	-5.68	-5.06			
019	8.10	8.74	7.61	8.47	9.26	7.89	8.87	9.45	8.62	-5.82	-6.18	-5.62	-6.35	-6.65	-5.87	-6.54	-6.67	-6.45
012	7.62	8.77	7.21	7.92	8.69	7.54	7.60	8.04	7.38	-6.13	-6.45	-5.86	-6.29	-6.68	-6.03	-6.14	-6.32	-5.98
010	7.67	8.12	7.99							-6.05	-6.19	-5.93	-5.95	-6.48	-5.70			
002	7.56	7.96	7.24	7.92	8.26	7.60	7.27	7.67	6.93	-7.19	-7.37	-6.87	-7.13	-7.34	-6.65	-7.40	-7.61	-6.91
015	7.54	7.89	7.35	7.82	8.37	7.41	7.31	7.72	6.98	-6.85	-7.05	-6.68	-6.77	-7.18	-6.46	-7.04	-7.27	-6.73
023	7.94	8.61	7.62	7.54	7.77	7.29	6.56	7.00	6.13	-7.60	-8.00	-7.37	-7.24	-7.36	-6.81	-7.10	-7.45	-6.58
014																		
020	8.19	8.59	7.75	8.38	8.89	7.99	8.12	8.55	7.77	-5.84	-6.01	-5.71	-6.30	-6.57	-6.03	-6.19	-6.38	-5.97
013	8.60	9.26	8.24	8.64	9.09	8.18				-6.88	-7.19	-6.68	-7.18	-7.30	-6.82			
021	7.66	8.03	6.91	8.24	8.88	7.88	8.76	9.32	8.34	-7.38	-7.79	-7.03	-7.28	-7.53	-7.06	-7.30	-7.90	-7.05
029	7.47	7.97	7.02	7.97	8.54	7.60	7.54	8.21	7.12	-6.93	-7.18	-6.40	-7.42	-7.78	-7.20	-7.72	-8.19	-7.31
022				8.21	8.64	7.98				-6.76	-7.44	-6.55	-6.78	-6.94	-6.67	-6.76	-7.44	-6.55
042	8.56	8.80	8.37	7.93	8.37	7.63	8.13	8.64	7.65	-8.46	-8.51	-8.40	-6.31	-6.44	-6.18	-6.48	-6.77	-6.24
040	7.68	7.92	7.54	7.33	7.54	7.19				-7.19	-7.28	-7.09	-7.13	-7.24	-7.00			
028	7.70	8.09	7.47	7.61	7.93	7.42	7.08	7.81	7.40	-7.66	-7.86	-7.47	-7.66	-7.87	-7.48	-7.43	-7.67	-7.12
039	8.19	8.93	7.79	8.39	8.62	8.16	7.71	7.84	7.55	-7.65	-8.11	-7.35	-7.56	-7.77	-7.41	-6.85	-6.95	-6.77
027																		
031	7.64	7.96	7.41	7.79	8.15	7.55	7.66	7.90	7.38	-7.52	-5.69	-7.29	-7.77	-7.98	-7.56	-7.27	-7.57	-7.09
034																		
043																		
049																		
018																		
001																		
046																		
041																		
024																		
038																		
004	8.11	8.69	7.70	7.68	8.15	7.38	8.03	8.66	7.64	-7.88	-8.27	-7.62	-7.58	-7.84	-7.35	-8.16	-8.53	-7.86
003	8.84	9.32	8.29	8.60	8.81	8.40	8.97	9.26	8.63	-5.84	-6.07	-5.35	-5.79	-5.86	-5.72	-5.74	-5.82	-5.47
050	8.15	8.62	7.81	8.67	9.06	8.33				-5.22	-5.35	-5.05	-5.65	-5.74	-5.41			
011	7.61	8.17	7.15	8.38	8.81	8.05	8.94	9.34	8.53	-5.56	-5.96	-5.26	-6.33	-6.50	-5.87	-6.66	-6.96	-6.52
016	7.24	8.44	6.94	8.06	8.53	7.80				-4.31	-5.05	-4.01	-5.26	-5.57	-5.05			
017	7.53	7.81	7.34				8.41	8.90	7.90	-6.55	-6.74	-6.37				-6.66	-6.90	-6.43
007																		
006	8.49	8.90	8.14	8.65	8.88	8.45	8.20	8.53	7.94	-5.73	-5.90	-5.56	-5.85	-5.94	-5.79	-5.98	-6.23	-5.83
009	7.70	8.00	7.42	7.13	7.81	6.78	7.17	7.69	6.88	-7.39	-7.65	-7.22	-6.98	-7.35	-6.64	-7.06	-7.30	-6.78
008	7.91	8.54	7.56	7.61	8.04	7.38	7.72	8.61	7.41	-7.74	-8.05	-7.42	-7.56	-7.88	-7.06	-7.66	-8.01	-7.41
025	7.28	7.44	7.17	7.74	8.03	7.46	7.04	7.52	6.69	-7.25	-7.32	-7.14	-7.17	-7.28	-7.05	-7.30	-7.69	-6.89

All measured log_10_-transformed values are given, where available. Unit for K_A_: M^-1^. Unit for IgG concentration: M. Values are rounded to two decimal places for reasons of representation in the table.

### Visualization, and interpretation of measurements

**Timing: 30 min**

Apart from simply obtaining a concentration and affinity value for an interaction (which may be the final goal when characterizing a monoclonal antibody), the downstream applications of knowing these parameters is immense and has been, or could be, utilized in many ways. The affinity and concentration data in general, as exemplified here for SARS-CoV-2 (summarized in Table 1), provide an excellent opportunity for utilization in other exploratory studies such as factors involved in coagulation^11^ or population-based seroprevalence estimates,^12^ amongst many others. In the current dataset, we have combined the affinity values with demographic and clinical data as well as with ELISA-derived immunoglobulin sub- and isotype titer values (shown as -log(EC_50_) or p(EC_50_) values), for multiple overlapping and distinct SARS-CoV-2 spike and nucleocapsid (NC) epitopes,^1^ part of which is shown in Table 2.

***Note:*** Determining the affinity of antibodies for the SARS-CoV-2 NC protein (instead of spike) could be another interesting approach. However, we have not performed MAAP assays using the NC in this protocol. Instead, we use information from ELISA for correlation analyses.

**Table 2:** Additional features of patients, some of which are used for exploratory data analysis

Groups	Patient number	Sex	Age group	REGN-COV treatment	wild type	delta	omicron
Non-infected/non-vaccinated	026	M	51–60	–	1.45	1.72	1.39
	037	F	51–60	–	0.65	1.18	0.12
Infected/non-vaccinated	005	F	71–80	Yes	6.01	5.85	2.14
	019	F	31–40	–	3.58	4.07	3.68
	012	M	61–70	–	3.36	4.05	3.41
	010	F	61–70	–	3.35	4.00	2.96
	002	M	61–70	–	3.30	4.34	2.46
	015	M	51–60	–	3.11	3.75	2.92
	023	F	51–60	–	2.51	3.43	2.41
	014	M	41–50	–	2.25	2.57	1.96
Non-infected/vaccinated	020	F	61–70	–	4.23	3.76	3.54
	013	M	71–80	–	3.49	3.42	3.45
	021	M	51–60	–	3.04	3.39	3.02
	029	F	71–80	–	3.04	3.16	2.21
	022	M	51–60	–	3.00	3.84	3.40
	042	F	71–80	–	2.93	3.46	3.36
	040	M	>80	–	2.70	3.08	2.51
	028	M	21–30	–	2.48	2.78	2.47
	039	M	71–80	–	2.37	2.99	2.66
	027	F	71–80	–	2.23	2.80	2.04
	031	M	61–70	–	2.15	2.78	2.38
	034	F	>80	–	2.13	2.69	2.05
	043	M	>80	–	2.09	2.51	2.30
	049	F	51–60	–	2.09	2.35	1.68
	018	M	51–60	–	2.00	2.12	1.74
	001	M	51–60	–	1.98	2.42	1.62
	046	M	51–60	–	1.86	1.98	1.50
	041	M	71–80	–	1.64	2.11	1.81
	024	F	41–50	–	1.53	2.05	1.61
	038	F	51–60	–	1.08	1.85	1.11
Infected/vaccinated	004	F	71–80	–	4.32	3.23	2.40
	003	M	>80	Yes	4.24	5.85	4.21
	050	M	71–80	Yes	4.20	4.06	1.39
	011	M	31–40	–	4.06	4.07	3.63
	016	M	61–70	Yes	4.02	5.85	1.24
	017	M	>80	–	3.83	3.99	3.68
	007	F	>80	–	3.83	5.85	4.23
	006	M	41–50	Yes	3.78	3.99	3.11
	009	F	71–80	–	3.06	4.06	2.75
	008	M	51–60	–	2.79	3.01	2.76
	025	F	>80	–	2.61	3.62	2.40

Sex: male (M) or female (F). Age was partitioned into age groups for this table. The p(EC_50_) values of IgG against wild type, delta, and omicron RBD are shown, rounded to two decimal places. More features of the same dataset are part of a manuscript recently published.^1^

Prior to the visualization and interpretation of the data it needs to be brought into an appropriate format.

30. We load the required libraries for the next steps in *R* statistical software, and we read the data presented in Tables 1 and 2 which were exported as CSV files.

library(gplots)

library(ggplot2)

library(ggpubr)

library(ggridges)

require(graphics)

library(tidyverse)

library(tidyr)

library(magrittr)

library(dplyr)

MAAP <- read.csv("MAAP.csv") # the first data frame

Additional <- read.csv("Additional.csv") # the second data frame

Combined_df <- left_join(MAAP,Additional,by="ID")

***Note:*** These steps are not pivotal to the determination of affinity and concentration (as it was generated by the Fluidity Cloud (Fluidic Analytics)), which constitute the key element of this protocol. While we provide code and examples for data exploration, we do not detail how to install or utilize *R* software (the code deployed here was written and executed in *R* 4.2.0 within *R Studio* 2022.07.1 Build 554). Similar plots to those that will be shown could be generated using other programming languages like *Python* or software packages like *GraphPad Prism*.

generate_colnames <- function(starts_with, ends_with){

 pattern <- paste0("ˆ", starts_with, ".∗", ends_with, "$")

 colnames <- colnames(Combined_df)[grepl(pattern, colnames(Combined_df))]

 colnames

}

starts_with_vec <- c(rep("KA", 3), rep("Conc", 3), "RBD")

ends_with_vec <- c(rep(c("(omicron|delta|wt)", "u", "l"), 2), "IgG")

all_columns_list <- mapply(starts_with = starts_with_vec, ends_with = ends_with_vec, FUN = generate_colnames,

         SIMPLIFY = FALSE, USE.NAMES = FALSE)

make_tidy_df <- function(columns, valuesTO){

 c <- Combined_df %>% dplyr::select(ID, all_of(columns), Groups, Sex, Age, REGN) %>%

  pivot_longer(cols = columns,

   names_to = "Type_sort",

   values_to = valuesTO) %>%

  mutate(Type_sort = case_when(str_detect(Type_sort, "delta") ∼ "delta",

        str_detect(Type_sort, "omicron") ∼ "omicron",

        TRUE ∼ "wt"))

}

all_tidy_dfs <- mapply(columns = all_columns_list, valuesTO = c("KA", "KA_u", "KA_l", "Conc", "Conc_u", "Conc_l", "ELISA"),

      FUN = make_tidy_df, SIMPLIFY = FALSE)

MAAP_ELISA_RBDs <- all_tidy_dfs %>% purrr::reduce(left_join, by = c("ID", "Groups", "Sex", "Age", "REGN", "Type_sort"))

MAAP_ELISA_RBDs <- MAAP_ELISA_RBDs %>%

 mutate(Type_sort_numbered = case_when(str_detect(Type_sort, "delta") ∼ "02_delta",

          str_detect(Type_sort, "omicron") ∼ "03_omicron",

          TRUE ∼ "01_wt"))

31. We then convert the data from the so-called wide into the long format, a requirement to perform some of the analyses in *R* with the libraries we prefer.

***Note:*** The *R* object called *MAAP_ELISA_RBDs*, which is the data frame used for all subsequent analyses, can be accessed here,^10^ together with the actual script. Script and *R* object, together, allow to conduct the analyses shown next.

Using the measured *K*_*A*_ and antibody concentration from the MAAP assay, we now present both these parameters from all patients in one plot.

**Figure 4:**
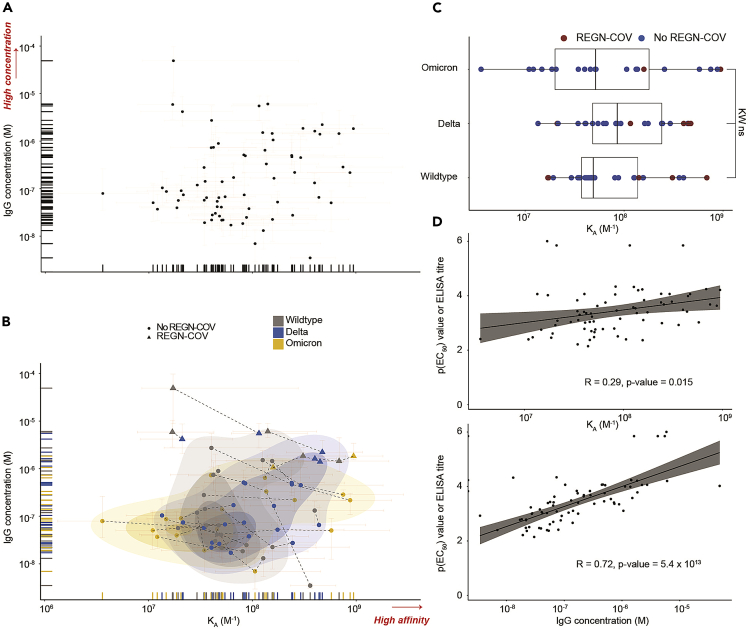
Data visualization and exploration (A) All quantifiable data points reflecting *K*_*A*_ (in M^−1^) and IgG concentration values (in M) are plotted.. (B) Same as (A) but including a 2D scatter plot with integrated density contours. Triangles denote patients receiving the REGN-COV cocktail. RBD variants: wild type (WT, gray), delta (blue), omicron (yellow). Dotted lines represent the measurements of the same patient sample against different RBD variants. Plot taken from.^1^ Higher affinity and higher concentration (for A and B) are indicated in red writing with an arrow. 95% confidence intervals for each point are colored in light red. (C) Boxplot analysis of *K*_A_ values for WT, delta, and omicron RBD variants. Samples of patients treated with REGN-COV antibody cocktail are shown in red color. (D) *K*_*A*_ and IgG concentration were plotted against the respective ELISA titers, i.e., p(EC_50_) values, obtained for WT, delta, and omicron RBD variants. Concentration, but not affinity, is shown to correlate with titers measured by ELISA. Data and plots are from work recently published.^1^

32. We execute the code to obtain a simple affinity versus antibody concentration plot, including the confidence intervals of the measurements (Figure 4A).

ggscatter(MAAP_ELISA_RBDs, x = "KA", y = "Conc",

  fullrange = TRUE,

  rug = TRUE) +

 ggtitle('KA versus IgG concentrations – all data') +

 labs(y="IgG concentration (M)", x='KA (1/M)') +

 geom_errorbar(data=MAAP_ELISA_RBDs, aes(ymin = Conc_l,

          ymax = Conc_u), size=0.05, width = 0.05, alpha=0.1, color = 'red') +

 geom_errorbarh(data=MAAP_ELISA_RBDs, aes(xmin = KA_l,

         xmax = KA_u), size=0.05, height = 0.05, alpha=0.1, color = 'red') +

 yscale("log10", .format = TRUE) +

 xscale("log10", .format = TRUE)

***Note:*** Here we plot the equilibrium association constant *K*_*A*_, which is the inverse of the equilibrium dissociation constant, *K*_*D*_. That is, the lower the *K*_*D*_ value the higher the affinity of the interaction. Both *K*_*A*_ as well as *K*_*D*_ can be used as a parameter to highlight the affinity of an interaction.

Next, we combine the MAAP data with additional information from Table 2. In this example, we aim to identify whether in this collective of patients with a history of infection with wild type or delta SARS-CoV-2 and/or vaccination with pre-omicron vaccines, the affinity of plasma antibodies statistically differs among the three RBD VOC.

33. We execute the code to obtain another version of the same plot, this time including information on the SARS-CoV-2 RBD antigen used (i.e., wildtype, delta, or omicron RBD) and whether the patients were therapeutically treated with monoclonal antibodies targeting SARS-CoV-2 spike (referred to as REGN-COV), see Figure 4B.

ggscatter(MAAP_ELISA_RBDs, x = "KA", y = "Conc",

  color = "Type_sort",

  shape = "REGN",

  palette = "jco",

  fullrange = TRUE,

  rug = TRUE) +

 stat_density_2d(data=MAAP_ELISA_RBDs, geom = "polygon",

    aes(alpha = ..level.., fill = Type_sort),

    bins = 4) +

 scale_alpha_continuous(range=c(0.1,0.3)) +

 ggtitle('KA versus IgG concentration - annotated') +

 labs(y="IgG concentration (M)", x='KA (1/M)') +

 geom_errorbar(data=MAAP_ELISA_RBDs, aes(ymin = Conc_l,

              ymax = Conc_u), size=0.05, width = 0.05, alpha=0.1, color = 'red') +

 geom_errorbarh(data=MAAP_ELISA_RBDs, aes(xmin = KA_l,

        xmax = KA_u), size=0.05, height = 0.05, alpha=0.1, color = 'red') +

 yscale("log10", .format = TRUE) +

 xscale("log10", .format = TRUE)

***Note:*** As mentioned earlier, there is a degree of samples where quantification was not possible as antibody concentration and/or affinity were below the limit of detection. This can be potentially important when interpreting the data as some data points cannot be shown. To manage such results, we have performed additional analyses and provided further explanations elsewhere.^1^

34. We then execute the code to look at the affinity differences between wild type, delta, and omicron RBD in a more statistical manner (Figure 4C).

ggscatter(MAAP_ELISA_RBDs, x = "KA", y = "Type_sort_numbered",

 conf.int = TRUE,

 color = "REGN",

 palette = c("#0073C2FF", "#A73030FF"),

 fullrange = TRUE,

 rug = FALSE,

) +

 ggtitle('KA versus Wildtype, Delta, Omicron') +

 labs(y="RBD variant", x='KA (1/M)') +

 geom_boxplot(alpha=0) +

 xscale("log10", .format = TRUE)

***Note:*** For statistical analyses, we first perform a Kruskal-Wallis test with subsequent Wilcoxon rank sum test with Holm correction for multiple comparisons, comparing groups with α < 0.01 for Kruskal-Wallis against all other groups.

35. We now aim to correlate both affinity and concentration with the respective ELISA-derived titer values for the same RBD variant. We execute the code to obtain a correlation plot (Figure 4D).

ggscatter(MAAP_ELISA_RBDs, x = "KA", y = "ELISA",

 add = "reg.line",

 conf.int = TRUE,

 fullrange = TRUE,

 rug = FALSE) +

 stat_cor() +

 ggtitle('KA versus ELISA titre') +

 labs(y="p(EC50) values", x='KA (1/M)') +

 xscale("log10", .format = TRUE)

 ggscatter(MAAP_ELISA_RBDs, x = "Conc", y = "ELISA",

 add = "reg.line",

 conf.int = TRUE,

 fullrange = TRUE,

 rug = FALSE) +

 stat_cor() +

 ggtitle('IgG concentration versus ELISA titre') +

 labs(y="p(EC50) values", x='IgG concentration (M)') +

 xscale("log10", .format = TRUE)

The Pearson correlation coefficient (R) for the correlation of ELISA p(EC_50_) values with *K*_*A*_ is 0.29 (p-value = 0.015), and with IgG concentration 0.72 (p-value = 5.4 × 10^−13^), indicating that ELISA titers (a mesh of affinity and concentration) are determined by antibody concentrations and much less by antibody affinity.

***Note:*** For further examples on how to correlate the different data types, including immunoglobulin sub- and isotypes, please refer to the following manuscript^1^ published in iScience.

## Expected outcomes

By following this protocol, we first labeled our antigen of interest (Figure 1), here the wild type, delta, and omicron variants of RBD from SARS-CoV-2 spike. The conjugation of a fluorophore to the probe protein is critical to the success of all remaining steps for affinity determination. We then used the labeled antigen to iteratively perform a MAAP assay for each patient sample and RBD antigen by using the Fluidity One-M (Fluidic Analytics) (Figure 2), which allowed us to determine both the affinity and concentration for each interaction (Figure 3 and Table 1). Finally, beyond its use as a biophysical description of a molecular interaction, as an example on what one can do with affinity data, we combined the affinity measurements with demographic information and ELISA-derived titers (see Table 2) for data exploration (Figure 4).

## Limitations

### Clonal diversity of the humoral immune response

Natural immune responses are commonly polyclonal,^13^^,^^14^^,^^15^^,^^16^^,^^17^^,^^18^ targeting multiple epitopes at different affinities. This apparent redundancy constitutes part of the breadth of immunity to generate a potent and robust antibody response that may be effective against multiple viral clades which collectively may provide resistance to antigen drift. The MDS-based methodology presented in this protocol is not, at the moment, capable of capturing the clonal diversity of the immune response. While reliable estimates of antibody concentration and affinity can be made, we have shown that they typically represent the strong binders within a polyclonal response.^2^ To delineate clonal diversity, targeted B-cell receptor repertoire sequencing,^19^^,^^20^ possibly combined with mass spectrometry-based approaches^21^ may be performed. These approaches are comprehensive, technically challenging, expensive, and descriptive and mandate the expression of monoclonal antibodies for subsequent characterization of affinity and function.^2^ As an intermediate, cost-effective, and easily accessible methodology, the combination of MAAP-based affinity data with ELISA-derived epitope characterization and immunoglobulin iso- and subtyping is recommended, as shown.^1^

### Antibody affinity and functional implications

The protocol outlined here aims to provide a guide on how to measure affinity and concentration (1) without the requirement for immobilization (i.e., the measurements conducted here are *in solution*), and (2) in complex biological samples like plasma. However, affinity and concentration alone are insufficient to unambiguously assert functionality. Yet, in the present case, affinity measurements correlated well with *in vitro* pseudotyped virus neutralization assays which give context to functionality.^3^ In addition to obtaining an affinity and concentration fingerprint, the MDS-approach is adaptable to other conceptual variations: for instance, we have previously used fluorescently labeled ACE2 complexed with SARS-CoV-2 spike S1 domain and employed a pre-incubation in the presence of patient plasma at varying concentrations. High-affinity binding of anti-S1 domain antibodies contained in plasma of seropositive individuals resulted in the disassembly of the ACE 2 – S1 complex in a dose-dependent manner, thereby resulting in a reduction of the *R*_*h*_ which was subsequently analyzed by means of MAAP assay.^2^^,^^22^

### Throughput of the methodology

The methodology presented in this protocol, using the Fluidity One-M device (Fluidic Analytics), allows completion of 3–4 independent MAAP assays in one working day (using one device), provided all reagents are available. Although this methodology cannot currently compete with large-scale approaches that require throughput of hundreds or thousands of samples per day,^12^ this number is cutting-edge from the perspective of a biophysical laboratory.

## Troubleshooting

### Problem 1

The degree of labeling is lower than 50%. A low degree of labeling reduces the fluorescence signal recorded by the Fluidity One-M at low protein concentrations, and thus reduces the dynamic range of MAAP in respect to high affinity interactions as these require measurement at low concentrations of probe protein (related to step 8).

### Potential solution

Check all protein and label concentrations used in the labeling mix, the pH of the NaHCO_3_ labeling buffer, and use a fresh, unopened vial of DMSO. Also analyze the size-exclusion chromatogram and measure the size of the labeled probe protein on the Fluidity One-M to check for protein aggregation. Protein aggregation can potentially block reactive groups available in the monomeric form of the protein. If no aggregation can be detected, repeat the labeling reaction with fluorescent label, fresh labeling buffer, and fresh DMSO after having checked that all concentrations are correct.

### Problem 2

Measured sizes after conjugation of fluorescent dye are more than 10% smaller than expected (related to step 9).

### Potential solution

This is likely caused by insufficient removal of unconjugated fluorescent label. For unconjugated fluorescent label, repeat chromatographic purification as described in step 6. A quick alternative to remove small amounts of unconjugated fluorescent label are spin columns such as Zeba™ Spin Desalting Columns, 7K MWCO, 0.5 mL which can be used in accordance with the manufacturer’s instructions.

### Problem 3

Complex formation is observed at every sample dilution (related to steps 28 and 29).

### Potential solution

This is typically caused by high levels of tightly binding antibodies in the sample. To resolve this issue, dilute the sample by at least a factor of ten and repeat the experiment. If experiments still do not converge using the Fluidity Cloud analysis tool – even at the lowest possible concentrations of probe protein – the affinity is too high and only an upper limit for *K*_*D*_ can be provided. Note that in many of such cases the antibody concentration is well-constrained and can be determined with high confidence.

### Problem 4

All measurements display the same size as free labeled probe (related to steps 28 and 29).

### Potential solution

The antibody affinity or concentration is too low to measure binding. If possible repeat measurements, at lower concentrations of labeled probe protein. If experiments still do not converge using the Fluidity Cloud analysis tool – even at the lowest possible concentrations of probe protein – the affinity or antibody concentration is too low to be quantified.

### Problem 5

Technical replicates (same combination of probe concentration and sample dilution measured repeatedly) differ more than 10% in size (related to steps 28 and 29).

### Potential solution

This can happen if the fluorescence intensity of the probe protein is lower than or equal to the background fluorescence of the serum or plasma in the absence of probe protein. Ensure that the labeled probe protein is used at concentrations that result in fluorescence intensities at least a factor of two higher than the signal of the background. Note that poor labeling efficiency as described in problem 1 of this troubleshooting section can contribute to low signal over background.

## Resource availability

### Lead contact

Further information and requests for resources should be directed to and will be fulfilled by the lead contact, Marc Emmenegger (marc.emmenegger@usz.ch).

### Materials availability

No new materials have been generated in this protocol.

## Data Availability

• Data used in this protocol has been generated and analyzed in a previous work, see.^1^ The dataset for analyses included in this protocol (chapter: Visualization, and interpretation of measurements) is publicly available on Zenodo^10^ and the DOIs are listed in the key resources table. • Code used in this study is publicly available on Zenodo^10^ and the DOIs are listed in the key resources table. • Any additional information required to reanalyze the data reported in this paper is available from the lead contact upon request.
